# Hemolymph-Mediated Dynamics of Paralytic Shellfish Toxins and Tetrodotoxin in Scallops

**DOI:** 10.3390/md24060200

**Published:** 2026-06-05

**Authors:** Ranmaru Matsui, Yuko Cho, Yuta Kudo, Keiichi Konoki, Kazue Nagasawa, Mari Yotsu-Yamashita

**Affiliations:** 1 Graduate School of Agricultural Science, Tohoku University, 468-1 Aramaki-Aza-Aoba, Aoba-ku, Sendai 980-8572, Japan; matsui.ranmaru.p8@alumni.tohoku.ac.jp (R.M.); yuko.cho.a4@tohoku.ac.jp (Y.C.); yuta.kudo.d5@tohoku.ac.jp (Y.K.); keiichi.konoki.b2@tohoku.ac.jp (K.K.); kazue.nagasawa.d6@tohoku.ac.jp (K.N.); 2 Frontier Research Institute for Interdisciplinary Sciences, Tohoku University, 6-3 Aramaki-Aza-Aoba, Aoba-ku, Sendai 980-8578, Japan

**Keywords:** scallop, paralytic shellfish toxins, tetrodotoxin, hemolymph

## Abstract

To investigate the dynamics of paralytic shellfish toxins (PSTs) and tetrodotoxins (TTX) in scallops, PSTs and TTX were analyzed in the hemolymph supernatant (hemolymph-S) and digestive gland in Yesso and Akazara scallops cultured in eastern Japan. In Yesso scallops sampled between 22 April 2025 and 21 October 2025, the PST concentrations in the hemolymph-S were 1.6–17% of those in the digestive gland, showing a positive correlation (*r* = 0.753). However, the PST composition in the hemolymph-S and digestive gland differed; the hemolymph-S composition initially resembled that of laboratory-cultured dinoflagellates, coinciding with typical onset of toxic dinoflagellate blooms in late April. Following this period, the PST composition in the hemolymph-S gradually converged with that of the digestive gland, via chemical transformation in the digestive gland followed by release into the hemolymph. The ratios of 11β-OSO_3_H toxins (C2, GTX4, GTX3) to 11α-OSO_3_H toxins (C1, GTX1, GTX2), which exist in chemical equilibrium, exhibited a similar trend. TTX was detected in both tissues of Yesso and Akazara scallops collected from 28 August 2025 to 27 January 2026, with the hemolymph-S concentration being 0.5–5.4% of that in the digestive gland. These results suggest that these toxins are sequestered into the digestive gland from the PST-producing dinoflagellates and certain TTX-bearing organisms, and then they partially flow into the hemolymph for circulation throughout the body.

## 1. Introduction

**Figure 1:**
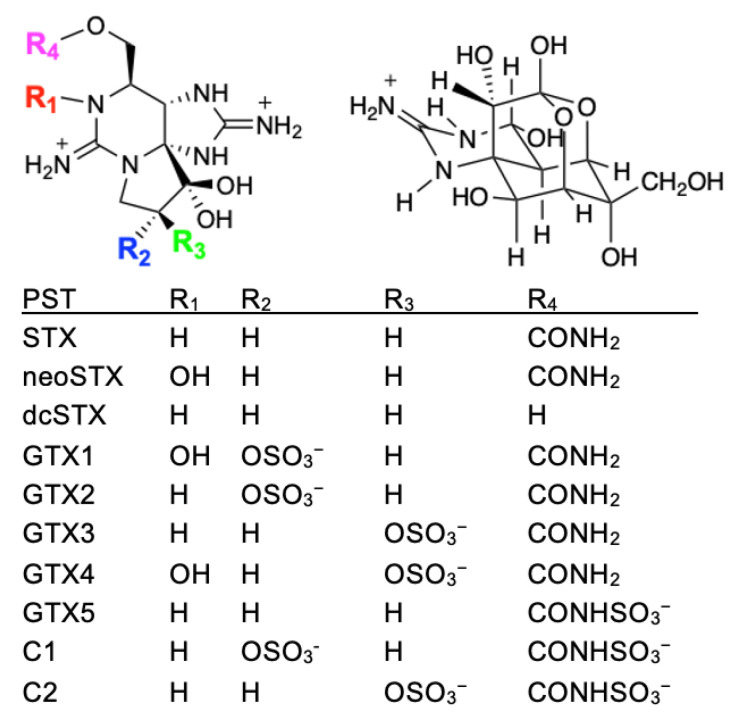
The structures of the PSTs and TTX analyzed in this study.

Paralytic shellfish toxins (PSTs) [1,2] and tetrodotoxin (TTX) [3,4,5,6] (Figure 1) are potent and specific voltage-gated sodium channel blockers [7,8]. Comprising more than 50 analogues, PSTs are typically classified into saxitoxins (STXs), including STX, neoSTX, and dcSTX, gonyautoxins (GTX1-6), which possess 11-OSO_3_H or 13-carbamoyl N-SO_3_H groups, C-toxins (C1-C4), which possess both 11-OSO_3_H and 13-carbamoyl N-SO_3_H groups [9], and M-toxins, which possess 11-OH or 11,11-dihydroxy groups [10,11]. PSTs are produced by specific marine dinoflagellate species, primarily within the genera *Alexandrium* [12] and *Gymnodinium catenatum* [13], and they are accumulated by various bivalves, including economically significant scallops, oysters, and mussels [1]. PSTs in seafoods are globally regulated to prevent potentially fatal food poisonings [14]. Recently, biosynthetic studies of PSTs have progressed significantly [15,16,17,18,19,20,21,22,23,24,25,26,27,28,29,30,31]. Additionally, TTX, which is known as pufferfish toxin, is generally detected at low concentrations in bivalves across Europe [32,33,34,35], New Zealand [36], China [36], and Japan [37,38,39,40,41]. The European Food Safely Authority (EFSA) established a regulatory threshold for TTX in seafoods at 44 µg/kg [42]. The origin of TTX in bivalves has not been clearly determined.

In eastern Japan, bivalves typically exhibit high PST concentrations, particularly in the digestive gland, from the middle of April to late July during extensive PST-producing dinoflagellates blooms. In this region, *Alexandrium* spp. typically begin blooming from the middle of April and generally approach the end of bloom by October [37,43]. Conversely, previous studies indicate that TTX accumulation in scallops peaks from late August to late October or November [37,41].

Li et al. [44] reported on the scallop genome and proposed the biotransformation of PSTs in scallop tissues; Liu et al. [45] and Li et al. [46] also reported biotransformation of PSTs in different tissues of Yesso scallops. These studies indicated the implication of the digestive gland and kidney in biotransformation. However, analytical data for PSTs and TTX in hemolymphs, which should be involved in the circulation of toxins, have never been reported.

In this study, to enable the further investigation of these dynamics, we focused on the circulation and metabolism of these toxins in scallops, and the concentration and composition of PSTs and TTX in the supernatant of the hemolymph (hemolymph-S) and digestive gland of two scallop species, *Mizuhopecten yessoensis* (Yesso scallop) and *Chlamys farreri akazara* (Akazara scallop)*,* cultured in eastern Japan. As the hemolymph in invertebrates is functionally comparable to the blood and lymph of vertebrates [47,48], this study is the first to analyze these toxins within the circulating body fluids of scallops.

## 2. Results

### 2.1. Determination of Protein Concentrations in Hemolymph-S

The protein concentrations of the hemolymph-S from two specimens of Yesso scallops collected on 22 April 2025 (approximately 10 mL/specimen) were determined to be 3.3 and 3.8 mg/mL. Similarly, the protein concentrations of the hemolymph-S (approximately 1 mL/specimen) collected from two specimens of Akazara scallops on 27 January 2026 were determined to be 2.3 and 2.4 mg/mL.

### 2.2. Analysis of PSTs and TTX in the Supernatant of Hemolymph (Hemolymph-S) of Yesso and Akazara Scallops

#### 2.2.1. Identification of PSTs in the Hemolymph-S of Yesso Scallops

**Figure 2:**
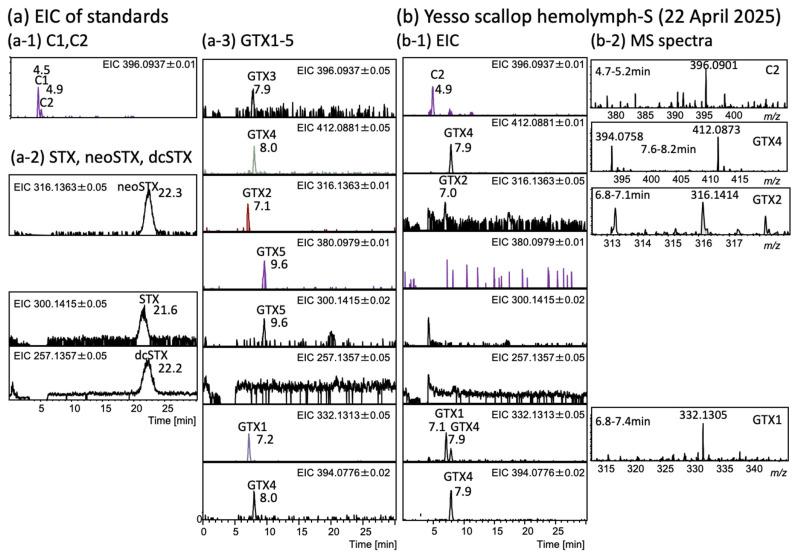
Identification of the major PSTs in the hemolymph-S of a Yesso scallop using HR-HILIC LCMS: (**a**) Extracted ion chromatograms (EICs) of the standard mixtures (**a-1**) C1 (0.978 µM) and C2 (0.218 µM) 1 µL on column, (**a-2**) STX (0.900 µM), neoSTX (1.954 µM) and dcSTX (0.56 µM) 1 µL on column, (**a-3**) GTX1 (1.520 µM), GTX2 (0.444 µM), GTX3 (0.154 µM), GTX4 (0.520 µM), and GTX5 (0.698 µM) 1 µL on column [49]; (**b**) EIC (**b-1**) and MS spectra (**b-2**) of PSTs in the hemolymph-S of a Yesso scallop collected on 22 April 2025. The retention time of the peak of each PST is shown near the peak.

Hemolymph obtained from five Yesso scallop specimens collected on 22 April 2025 (totaling approximately 30 mL) was separated into the hemolymph-S and a precipitate (approximately 0.1 g) containing blood cells via centrifugation for PSTs and TTX analysis. Following extraction of the precipitate with 0.2 M AcOH and subsequent charcoal treatment, PST and TTX concentrations were determined to be below the limit of detection (LOD, PSTs < 0.06 nmol/g, TTX < 0.04 nmol/g). To analyze PSTs, hemolymph-S (1 mL) was subjected to charcoal treatment. Based on the retention times and exact molecular MS determined using high-resolution (HR) hydrophilic interaction chromatography (HILIC) liquid chromatography mass spectrometry (LCMS) (Figure 2), PSTs (C2, GTX1, GTX2, GTX4) were identified, although the peak of GTX2 was below the limit of quantitation (LOQ).

#### 2.2.2. Identification of TTX in the Hemolymph-S of Yesso and Akazara Scallops

**Figure 3:**
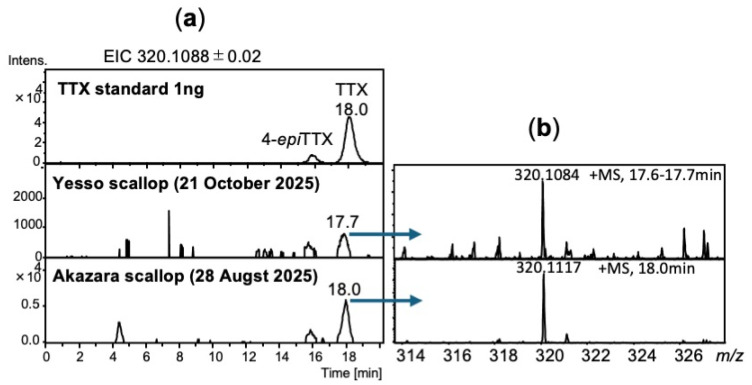
Identification of TTX in the hemolymph-S from Yesso and Akazara scallops using HR-HILIC-LCMS: (**a**) EICs detected at *m*/*z* 320.1088 ± 0.02; (**b**) MS spectra of TTX in a Yesso scallop (21 October 2025) and Akazara scallops (28 August 2025).

For the initial test, the hemolymph-S (total 1.2 mL) and a precipitate (approximately 50 mg) were obtained from 10 specimens of Akazara scallops collected on 16 September 2025. PSTs and TTX concentrations in the precipitate were below the LOD (PSTs < 0.06 nmol/g, TTX < 0.04 nmol/g). In hemolymph-S, TTX was detected (0.18 nmol/mL) using HILIC LCMS (multiple reaction monitoring; MRM mode) [50] (Figure S3), whereas the PST level was below the LOD (<0.06 nmol/mL). TTX was further identified using HR-HILIC LCMS [51] in the hemolymph-S of Yesso scallops collected on 21 October 2025 and from Akazara scallops collected on 28 August 2025 (Figure 3). TTX in the hemolymph-S of the 21 October 2025 Yesso scallop was below the LOQ of HR-HILIC LCMS. However, the [M+H]^+^ (*m*/*z* 320.1084) of TTX was accurately detected, and the dehydrated ion at *m*/*z* 302.0904 and the characteristic product ion of TTX at *m*/*z* 162.0614 [51] were detected in the LCMSMS spectrum of TTX (Figure S4) in this sample. LCMSMS spectra of TTX in the hemolymph-S of Akazara scallop (28 August 2025) and standard TTX are also shown in Figure S4 for comparison. These results support the identification of TTX. Based on these results, TTX was quantified only in the hemolymph-S in the subsequent experiments.

### 2.3. Quantitative Analysis of PSTs and TTX in the Hemolymph-S and Digestive Gland of Yesso and Akazara Scallops

The total concentrations of PSTs (C1/C2, GTX1-5, and STX, neoSTX and dcSTX) and TTX in the hemolymph-S and digestive gland of Yesso scallops (22 April 2025, 20 May 2025, 27 June 2025, and 21 October 2025) and Akazara scallops (28 August 2025 and 27 January 2026) were quantitatively analyzed using liquid chromatography–fluorescence detection (LC-FLD) for PSTs [49] (Figure S1) and LC-FLD [52,53] (Figure S2) and HILIC-LCMS (MRM) [50] for TTX (Figure S3). The results are summarized in Table 1. Details of the analytical data of the PSTs are given in Table S1.

**Figure 4:**
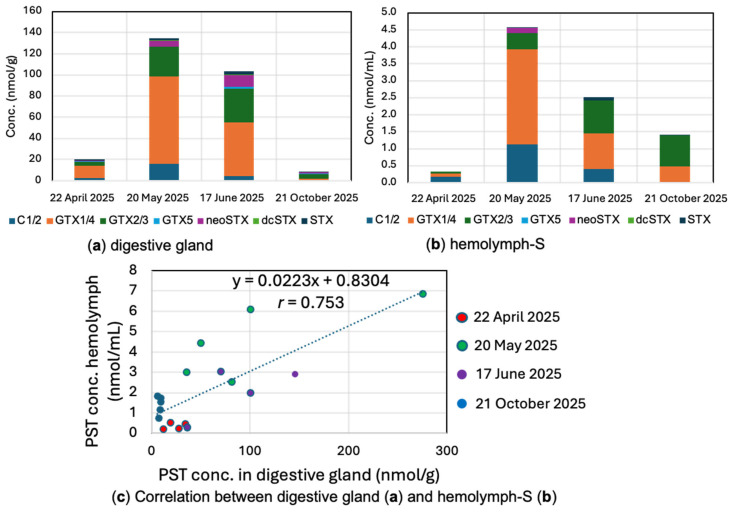
Comparison of PST concentration and composition between the digestive gland and hemolymph-S of Yesso scallops collected on 22 April 2025, 20 May 2025, 17 June 2025, and 21 October 2025; (**a**) digestive gland; (**b**) hemolymph-S (mean, *n* = 5 for each sampling time); (**c**) the correlation between total PST concentrations between digestive gland (**a**) and hemolymph-S (**b**) for all samples at the four sampling times (*n* = 20).

Figure 4a,b shows the PST concentrations in the hemolymph-S and digestive gland of Yesso scallop samples collected on four occasions, on 22 April 2025, 20 May 2025, 27 June 2025 and 21 October 2025. The PST concentrations in the hemolymph-S of the Yesso scallop samples were 1.6–17% (mol/mol) of those detected in the digestive gland. As shown in Figure 4c, the PST concentrations in the hemolymph-S and digestive gland were positively correlated (y = 0.0223x + 0.8304, *r* = 0.753), with x and y denoting the PST concentrations in the digestive gland (nmol/g) and hemolymph-S (nmol/mL), respectively.

In Akazara scallops, total PST concentrations in the hemolymph-S in all samples tested were below the LOQ (<0.06 nmol/mL), while PSTs were detected at low concentrations in the digestive gland. Regarding TTX, it was detected both in the hemolymph-S and digestive gland of Yesso scallops (21 October 2025) (Figure 3) and Akazara scallops (28 August 2025 (Figure 3) and 27 January 2026). The ratios of concentration of TTX in the hemolymph-S to that in digestive gland were 0.5–5.4% (mol/mol) (Table 1).

### 2.4. Comparison of PST Toxin Profiles Between the Hemolymph-S and Digestive Gland of Yesso Scallops, and Cultured Alexandrium catenella (Group I)

**Figure 5:**
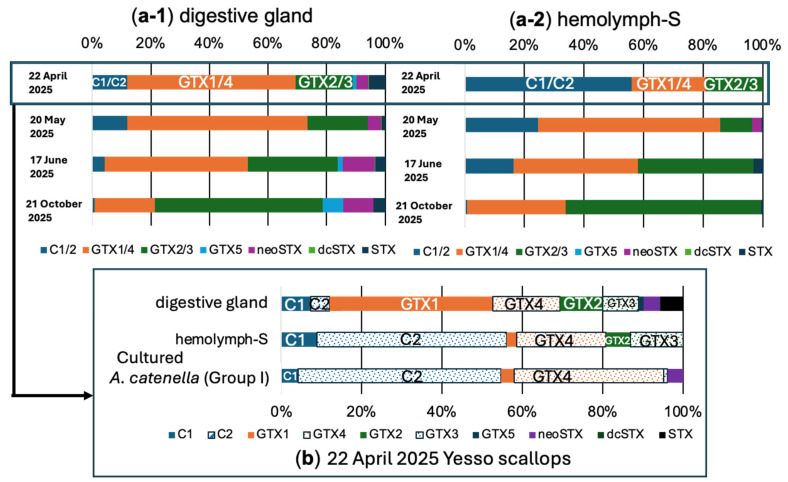
PST compositions in the digestive gland (**a-1**) and hemolymph-S (**a-2**) of Yesso scallops (mean, *n* = 5), and the detailed compositional profiles of Yesso scallops collected on 22 April 2025 compared with those of *A. catenella* (Group I) (**b**), which was isolated from a bay in eastern Japan and cultured in a laboratory. The *A. catenella* (Group I) data in **b** was adapted from published data [26].

Figure 5(a-1,a-2) shows PST compositions in the digestive gland and hemolymph-S from the Yesso scallops collected at four intervals between 22 April 2025 and 21 October 2025. In eastern Japan, *A. catenella* (Group I) typically begins blooming on approximately 22 April, while the bloom of PSTs producing dinoflagellates generally approaches its end by 21 October [37,43]. Notably, the PST compositions in the digestive gland and hemolymph-S in 22 April 2025 differed more significantly than those observed at the other three sampling times (20 May 2025, 17 July 2025, 21 October 2025). In the 22 April 2025 samples, GTX1/4 (57%) and C1/C2 (12%) were the major and minor PSTs components in the digestive gland, respectively, whereas C1/C2 (56%) and GTX1/4 (25%) were the major and minor components in the hemolymph-S, respectively. In the 20 May 2025 samples, the composition of PSTs in the digestive gland and hemolymph-S resembled each other, showing that GTX1/4 is the major component in both tissues. In the hemolymph-S, neoSTX (3.3%) was only detected (>LOQ) in four out of the five samples collected on 20 May 2025, but not in the samples collected on the other three occasions. This is likely because the concentration of total PSTs in the 20 May 2025 samples is the highest among the four sampling times (Figure 4b). After 20 May 2025, the proportion of GTX2/3 gradually increased, while that of C1/C2 and GTX1/4 decreased both in the digestive gland and hemolymph-S. Regarding the minor components, the proportions of neo-STX, STX and GTX5 were increased in the digestive gland, although this trend was not observed in the hemolymph-S.

Figure 5b compares more detailed compositions of PSTs in the digestive gland and hemolymph-S collected on 22 April 2025 with the cellular extract of the dinoflagellate *A. catenella* (Group I), which was isolated from eastern Japan and cultured in a laboratory. This dinoflagellate is the primary PST-producing species in this region around late April. The PST profile of the hemolymph-S resembled that of *A. catenella* (Group I), with C2 as the major component, followed by GTX4, whereas the digestive gland profile appears distinct from that of *A. catenella* (Group I).

### 2.5. Comparison of the Proportions of 11β-OSO_3_H and 11α-OSO_3_H Toxins in the Digestive Gland and Hemolymph

**Figure 6:**
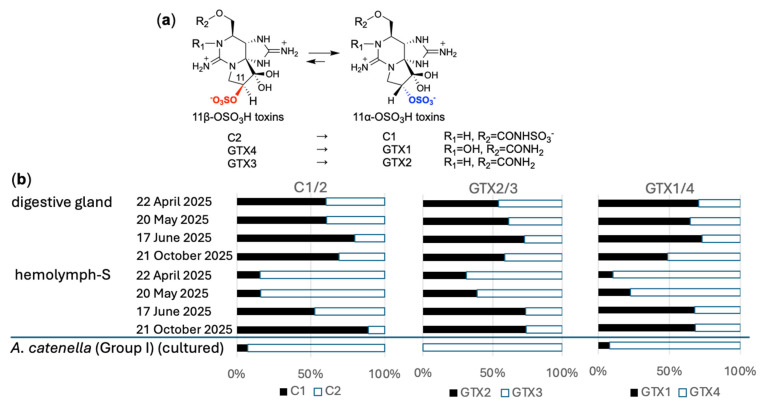
The seasonal variation in PSTs between 11β-OSO_3_H toxins and 11α-OSO_3_H toxins: (**a**) Chemical equilibrium of PSTs between 11β-OSO_3_H toxins and 11α-OSO_3_H toxins; (**b**) The proportions of C1/C2, GTX2/3, and GTX1/4 in the digestive gland and hemolymph-S of Yesso scallops (mean, *n* = 5) and that of the cellular extract of laboratory-cultured *A. catenella* (Group I). The *A. catenella* (Group I) data in (**b**) was adapted from published data [26].

Figure 6 compares the proportions of 11β-OSO_3_H toxins (C2, GTX4, GTX3) and 11α-OSO_3_H toxins (C1, GTX1, GTX2) (see Figure 1 for structures) in the digestive gland and hemolymph-S of four Yesso scallop samples. In the hemolymph-S samples from 22 April 2025 and 20 March 2025, 11β-OSO_3_H toxins (C2, GTX4, GTX3) were predominant over 11α-OSO_3_H toxins (C1, GTX1, GTX2). Conversely, 11α-OSO_3_H toxins (C1, GTX1, GTX2) were predominant in all the digestive gland samples and the hemolymph-S samples collected on 17 June 2025 and 21 October 2025. The profiles of the hemolymph-S from 22 April 2025 and 20 March 2025 were similar to that of the cellular extract from *A. catenella* (Group I), which was isolated in eastern Japan and cultured in a laboratory. This *A. catenella* (Group I) species primarily produces C2 and GTX4 as the major toxins [26].

## 3. Discussions

This study qualitatively and quantitatively analyzed PSTs and TTX in the hemolymph of bivalves for the first time, and it compared the results of these toxins with those detected in the digestive gland. These results provide significant insights into toxin dynamics, including the intake, transfer, accumulation in organs, and structural transformation of PSTs in scallops.

PSTs were detected in the hemolymph-S of Yesso scallops in all four samples collected between 22 April 2025 and 21 October 2025, whereas PSTs levels in the hemolymph-S in Akazara scallops collected on 28 August 2025 and 27 January 2026 were below the LOD (<0.06 nmol/mL) (Table 1). This absence of PSTs in Akazara scallops is attributed to the low cellular density of *A. catenella* (Group I) during these seasons in this region [37,43].

The PST concentrations in the hemolymph-S and digestive gland in Yesso scallops were positively correlated (Figure 4c) with the hemolymph-S concentrations representing 1.6–17% of those recorded in the digestive gland. In the cultivation region, PST-producing dinoflagellate *A. catenella* (Group I) typically exhibits intensive blooms from late April to mid-July [37,43]. According to the literature [43], *A. catenella* (Group I) cells increased from April to early May 2013, up to 136,200 cell/L, in a bay in eastern Japan. The correlation between the cellular density of *A. catenella* (Group I) in seawater with the PSTs concentrations in the digestive gland [37,43] suggests that PSTs originating from the toxin-producing dinoflagellates are ingested by the scallops and processed in the digestive gland, after which a specific portion enters the hemolymph circulation.

Regarding PST composition, C2 was the primary component in the hemolymph-S of scallops collected on 22 April 2025, followed by GTX4. This profile closely resembles that of *A. catenella* (Group I), but it differs from the digestive gland profile, where GTX1 was the dominant component (Figure 5). Late April typically marks the onset of increasing *A. catenella* (Group I) cellular density. At this stage, the original PST composition from the dinoflagellates was maintained as it enters the hemolymph. Following this period, the PST composition in the hemolymph-S gradually shifted to resemble that in the digestive gland, where GTX2/3 predominated. This suggests that PSTs that accumulate in the digestive gland undergo chemical transformation in this organ, from C2 to GTX2/3, via the hydrolysis of NH-SO_3_H to NH_2_ at the 13-carbamoyl group, followed by the release of GTX2/3 into the hemolymph.

Analysis of the chemically equilibrated C-11 epimers, 11β-OSO_3_H toxins (C2, GTX4, and GTX3) and 11α-OSO_3_H toxins (C1, GTX1, and GTX2) revealed that the hemolymph-S collected on 22 April 2025 and 20 May 2025 clearly contained a significantly higher proportion of 11β-OSO_3_H toxins than 11α-OSO_3_H toxins (Figure 6). This pattern differed from that of the digestive gland profile. In particular, in the 22 April 2025 sample, the hemolymph-S profile (dominated by 11β-OSO_3_H toxins such as C2 and GTX4) closely resembles that of the dinoflagellate culture shown in the same figure, while the digestive gland profile is different. The hemolymph-S profile then shifts over time. This strongly implies that the hemolymph-S receives freshly ingested toxins before they are structurally altered in the digestive gland. From 17 June 2025, the hemolymph-S profile transitioned to mirror that of the digestive gland. Due to their higher chemical stability [54], the 11β-OSO_3_H toxins produced by dinoflagellates spontaneously changed to 11α-OSO_3_H toxins. Therefore, from 17 June 2025, the hemolymph is likely supplied by PSTs that have already reached chemical equilibrium within the digestive gland. There appears to be a rapid biotransformation of β-epimers (C2, GTX4, and GTX3) to α-epimers (C1, GTX1, and GTX2) in the digestive gland, since, at time 0 (22 April 2025) in Yesso scallops, the toxin profile is already transformed with respect to the dinoflagellate *A. catenella*. This has already been documented for mussel tissues [55], suggesting that extracellular digestion in the stomach or intracellular digestion in the digestive gland may be the primary mechanism responsible. A similar result has been reported in the uptake kinetics of PSTs from *A. fundyense* in the mussel *Mytilus edulis* [55], and also in changes in PSTs in the gills and digestive glands of the cockle *Cerastoderma edule* under post-bloom natural conditions [56].

During the sample preparation procedure, the digestive gland was acidified with AcOH and heated in boiling water for 5 min, while the hemolymph-S was not acidified and not heated. The influence of these different treatments on PSTs composition was preliminary evaluated using hemolymph-S. The result (Figure S5) indicated that heating the sample in diluted AcOH caused partial transformations from C2 to C1 and GTX4 to GTX1, but transformations from C1/C2 to GTX2/3 (hydroxylation of carbamoyl *N*-SO_3_), or to dcSTX, neoSTX, and STX were not indicated. These results suggested that the ratios of C1/C2, GTX1/4, and GTX2/3 in the digestive gland in Figure 5b and Figure 6b were possibly influenced by this partial transformation during sample preparation. However, the total concentrations of C1+C2, GTX1+GTX4, GTX2+3, dcSTX, neoSTX, and STX were not significantly affected by this treatment.

TTX was detected in the hemolymph-S of the Yesso scallop samples collected on 21 October 2025 and Akazara scallop samples collected on 28 August 2025 and 27 January 2026 (Table 1, Figure 3), with the concentrations ranging from 0.5 to 5.4% of those in the digestive gland. The TTX concentrations in the Yesso scallops sampled between April and June 2025 remained below the LOD in both tissues. TTX analogues other than 4-*epi*TTX and 4,9-anhydroTTX were not detected (<0.04 nmol/mL), despite prior reporting of the detection of 5,6,11-trideoxyTTX in Akazara scallops [40]. The detection of TTX in scallops from late August to December in this region is consistent with our previous findings [37]. Notably, this is the first report of TTX detection in Akazara scallops collected in late January in this region. While the exact origin of TTX in scallops remains under investigation, Itoi’s group reported that they detected DNA of highly toxic flat worm *Planocera multitentaculata* in digestive glands of Yesso scallops [39]. Additionally, Dhanji-Rapkova et al. [57] suggested the ribbon worm *Cephalothrix simula* as a potential source of TTX in European bivalves.

**Figure 7:**
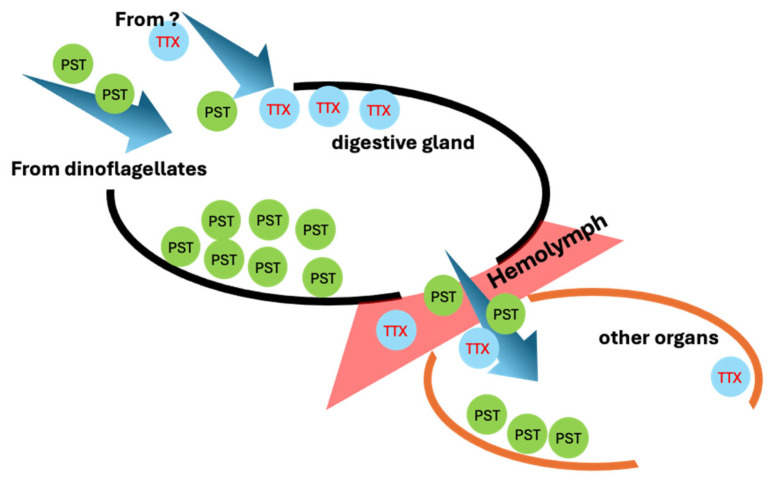
Hypothetical model of the PSTs and TTX dynamics based on the results of this study. Toxins are ingested and transported to the digestive gland for digestion, after which they partially enter the hemolymph for systemic circulation to various organs throughout the scallop body.

Li et al. reported that the scallop uses its digestive gland to accumulate PSTs and its kidney to transform them to high-toxicity forms through expanded sulfotransferases [44]. Based on the results of this study, together with their results, we propose the dynamics of PSTs and TTX as illustrated in Figure 7. During filter feeding, PSTs produced by dinoflagellates and TTX derived from certain organisms are ingested by scallops through the inhalant opening formed by the mantles, transported to the gills, and then delivered to the digestive gland for digestion. Subsequently, a portion of these toxins enters the hemolymph, while the remainder is partially accumulated in the digestive gland. The toxins within the hemolymph likely circulate systemically to other organs, including the kidneys, muscles, mantle, and gonads, before a fraction is excreted outside from the scallops. Hemolymph likely serves a role in the internal circulation and regulation of toxins throughout the scallop body. Zheng et al. recently reported the metabolic transformation of PSTs in the mussel, analyzing PSTs in the digestive gland, mantle, and other edible tissues [58]. In the current study, we analyzed PSTs and TTX only in hemolymph-S and the digestive gland. To collect more experimental evidence to support our proposed hypothetical model of toxin dynamics, the toxins in other organs should be analyzed in the next study.

## 4. Materials and Methods

### 4.1. Scallops

Living adult Yesso scallops (size 10.5–14.1 × 12.5–15.5 cm; collected on 22 April 2025, 20 May 2025, 17 June 2025, and 21 October 2025) and Akazara scallops (size 7.5–9.3 × 7.2–8.9 cm; collected on 28 August 2025 and 27 January 2026) cultured at sea depths of 10–30 m were purchased from a farm in eastern Japan. Specific geographic details of the region of the farm are withheld to prevent commercial impact. For all samples, the number of specimens was five (*n* = 5), except for the Akazara scallops collected on 28 August 2025 (*n* = 1). For the 28 August 2025 Akazara scallops, hemolymph was obtained from only two specimens (0.1 and 0.4 mL); these hemolymph samples were pooled for analysis (*n* = 1), and their corresponding digestive gland tissues were similarly pooled (*n* = 1). The size and weight of the collected scallops are summarized in Table S2.

### 4.2. Collection of Hemolymph and Preparation of Hemolymph-S, and Determination of Protein Concentrations of Hemolymph-S

The hemolymph was collected immediately upon the arrival of each specimen at the laboratory via withdrawal from the blood vessel in the smooth adductor muscle using a disposable syringe (5 mL) with a 22G or 23G needle (Terumo, Tokyo, Japan), without the use of modified Alsever’s solution [47,48]. The collected hemolymph volumes from each specimen ranged from 1 to 10 mL for Yesso scallops and 0.1 to 2.5 mL for Akazara scallops. The hemolymph-S was obtained via centrifugation at 13,200× *g* for 10 min at 4 °C. The protein concentration of hemolymph-S was measured to confirm that it contained protein, because the hemolymph of the scallops seemed to be just seawater. The protein concentration was determined using DC protein assay (Bio-Rad, Hercules, CA, USA) and Bovine Serum Albumin (Nacalai tesque, Kyoto, Japan) as a standard.

### 4.3. Sample Preparation of the Hemolymph-S and the Digestive Gland from Scallops for LC-FLD and LCMS Analysis

An aliquot (1 mL) of the hemolymph-S was neutralized with 0.5 M AcOH and applied to a charcoal column (1.5 mL) packed in a glass pipette. After washing with water (4.5 mL), toxins were eluted with AcOH/EtOH/H_2_O (5:50:45, *v*/*v*, 7.5 mL). The eluate was concentrated and then dissolved with 0.05 M AcOH (0.2 mL). Portions of this solution were then subjected to LC-FLD for PSTs (10 µL) [49] and HILIC-LCMS (MRM) for TTX (5 µL) [50]. This solution was also applied to HR-HILIC-LCMS for PSTs [59] and TTX [51] for further toxin identification (Figure 2 and Figure 3).

Similarly, digestive gland tissue was dissected out from each specimen, ground with a mortar and pestle with an equal volume of 1% aqueous AcOH (*v*/*v*), and heated in boiling water for 5 min. Following cooling to room temperature, the homogenate was centrifuged at 4000× *g* for 15 min at 4 °C. A portion of the supernatant (0.2 mL) was filtered through cotton and defatted with hexane (0.2 mL) in a glass microtube via vortexing and centrifugation at 1100× *g* for 1 min (repeated thrice). The aqueous layer was concentrated under vacuum, dissolved with 0.05 M AcOH (0.2 mL), and neutralized with 2.5% aqueous NH_4_OH (*v*/*v*), before being applied to a charcoal column (0.3 mL) packed in a glass pipette. After washing the charcoal with water (0.9 mL), the toxins were eluted with AcOH/EtOH/H_2_O (5:50:45, *v*/*v*, 1.5 mL), dried under vacuum, and then dissolved with 0.2 mL of 0.05 M AcOH. A 10 µL aliquot was analyzed for PSTs via LC-FLD. For TTX analysis via LC-FLD, the charcoal-purified solution was neutralized with 2.5% aqueous NH_4_OH (*v*/*v*) and applied to a BioRex70 weak cation resin (0.2 mL) (Bio-Rad). The resin was washed with water (1 mL), and the toxins were eluted with 0.2 M AcOH (2 mL). The eluate was concentrated, dissolved with 0.05 M AcOH (0.2 mL), and analyzed (10 µL) using TTX LC-FLD [52,53].

### 4.4. The Standards Solutions of PSTs and TTX

The standard solutions of PSTs were provided by Prof. Yasukatsu Oshima (Prof. emeritus of Tohoku University) [49]: TUKC92112 (1/50) C1 0.978 µM, C2 0.218 µM, TUMG913 1/50, GTX4 0.52 µM, GTX1 1.520 µM, GTX5 0.698 µM, GTX3 0.154 µM, GTX2 0.444 µM, TUMS913 (1/5) neoSTX 1.954 µM, dcSTX 0.530 µM, STX 0.900 µM. For the spike test of GTX1/4, GTX1/4 was purchased from CRM, National Research Council Canada. The standard solution of TTX (3.13 µM) was prepared as a mixture with major TTX analogues by the authors from the eggs of the pufferfish *Takifugu flavipterus* [51]. For these standards, no major contaminant peak was detected on total ion chromatograms using HR-HILIC-LCMS.

### 4.5. Spike and Recovery Tests

For the above sample preparation methods, spike and recovery tests were conducted using toxin-free Yesso scallops specimens (cultured on 9 March 2026, Nemuro, Hokkaido, Japan) by adding GTX1/4 (500 ng, CRM, National Research Council Canada) and TTX standards (500 ng, purified from pufferfish) to the hemolymph-S (1 mL) and digestive gland extract supernatant (0.2 mL). The recovery of GTX1/4 for charcoal purification was 100% (mean, *n* = 2) for the hemolymph-S and 90% (mean, *n* = 2) for the digestive gland using PST-LC-FLD. The recovery of TTX in hemolymph-S for charcoal purification was 51% (mean, n = 2) when analyzed via TTX LC-FLD, whereas that (same sample) analyzed using TTX HILIC-LCMS (MRM) yielded 31% (mean, *n* = 2) due to ion suppression. For the digestive gland, the recovery of TTX following sequential charcoal and BioRex70 purification was 51% (mean, *n* = 2) via TTX LC-FLD. BioRex70 treatment was necessary to eliminate the peaks overlapping TTX in the digestive gland matrix. Based on the difference in TTX recovery between LCMS (MRM) (31%) and LC-FLD (51%), the TTX concentrations in the hemolymph-S were compensated by a factor of 1.6 relative to that of the concentrations estimated via LCMS (MRM), as detailed in Table 1.

### 4.6. LC-FLD for PSTs and LC-FLD for TTX

PST LC-FLD was performed as reported by Oshima [49]. TTX LC-FLD was essentially performed as reported by Shoji et al. [53]. These methods utilize a charcoal pretreatment. TTX LC-FLD conditions were slightly revised as follows; Column: Develosil C30 UG-5 (5 µm, i.d. 0.46 × 15 cm), LC solvent: an aqueous solution containing 1 vol% acetonitrile, 20 mM ammonium heptafluorobutyrate, and 10 mM ammonium formate buffer (pH 5.0) at a flow rate of 0.4 mL/min using a pump Hitachi L-6000 (Tokyo, Japan). The eluted compounds were heated with 4 N NaOH (flow rate 0.7 mL/min, pump Hitachi L-7100) at 105 °C in a stainless tube (i.d. 0.46 mm × 5 m). The reaction products were detected using a Jasco FP2025 fluorescent detector with excitation 365 nm and emission 510 nm. A Hitachi D-2500 Chromato-Integrator was utilized for data acquisition and plotting.

The LOD (S/N = 5) using LC-FLD; PSTs C1 0.80, C2 0.72, GTX1 12, GTX2 3.0, GTX3 2.7, GTX4 18, GTX5 8.2, neoSTX 2.5, dcSTX 5.2, STX 5.3 (total PSTs 58) (pmol/mL or pmol/g), and TTX 280 pmol/mL or pmol/g. The LOQ (S/N = 10) of PSTs C1 1.6, C2 1.42, GTX1 24, GTX2 6.0, GTX3 5.4, GTX4 36, GTX5 16.4, neoSTX 5.0, dcSTX 10.4, STX 10.6 (total PSTs 116), and TTX 560 (pmol/mL or pmol/g). Representative LC-FLD chromatograms of PSTs and TTX are shown in Figures S1 and S2, respectively, in the Supporting Information (SI).

### 4.7. HILIC-LCMS (MRM) for TTX, and HR-HILIC-LCMS for PSTs and TTX

HILIC-LCMS (MRM) for TTX was performed using a column TSKgel amide-80 (i.d. 2.0 mm × 15 cm, 5 µm, Tosoh, Tokyo, Japan) with the following gradient system: A: 10 mM HCOONH_4_/H_2_O-HOOH (100:0.0005 *v*/*v*), B: MeCN, 0–10 min (A 75%, B 25%), 20–25 min (A 45%, B 55%), 27–29 min (A 35%, B 65%), 30–50 min (A 75%, B 25%). A 0.2 mL/min flow rate was employed. An API2000^TM^ mass spectrometer (SCIEX, Marlborough, MA, USA) was used in the MRM mode, with *m*/*z* 320/162, 302/162, 272/162, and 254/162, and the MS conditions for MRM were used as reported previously [50]. HR-HILIC-LCMS for PSTs [59] and TTXs [51] were performed using micrOTOFQII mass spectrometer (Bruker, Billerica, MA, USA) according to previously reported methods. The limit of TTX detection (S/N = 5) on the column using HILIC-LCMS (MRM) was previously reported as 64 pg [50]. The LOD (S/N = 5) and LOQ (S/N = 10) using HLIC-LCMS (MRM) for TTX for the hemolymph-S were 0.04 and 0.08 nmol/mL, respectively, and those for the digestive gland were 0.6 and 1.2 nmol/g, respectively. Representative HILIC-LCMS (MRM) mass chromatograms of TTX are shown in Figure S3.

## 5. Conclusions

PSTs and TTX were analyzed in the hemolymph of Yesso and Akazara Scallops cultured in eastern Japan. These toxins were detected in the supernatant of hemolymph (hemolymph-S) during the specific seasons when the concentrations of these toxins in the digestive gland are elevated. The concentrations of PSTs in the digestive gland and hemolymph-S of Yesso scallops are positively correlated. At the onset of dinoflagellate blooms, the PST composition in the hemolymph-S in Yesso scallops resembled that of PSTs producing dinoflagellates, but it differed from that observed in the digestive gland. Subsequently, the toxin profile of the hemolymph-S shifted to mirror that of the digestive gland. Based on these findings, a model for PSTs and TTX dynamics was proposed: a portion of the toxins ingested from external toxin-producing organisms directly and rapidly flows into the hemolymph. Another portion of the toxins is initially transported to the digestive gland for digestion, after which a portion enters the hemolymph to circulate throughout the scallop body.

## Figures and Tables

**Table 1:** The concentrations of total PSTs and TTX in the hemolymph-S and the digestive gland of the scallops.

		Total PSTs Concentration (Mean ± SD)						TTX Concentration (Mean ± SD)				
		(nmol/mL)		(nmol/g)				(nmol/mL)		(nmol/g)		
Species	Date	Hemolymph-S (A)	*n*	Digestive Gland (B)	*n*	A/B (%)		Hemolymph-S (A)	*n*	Digestive Gland (B)	*n*	A/B (%)
Yesso scallop	22 April 2025	0.33 ± 0.11	5	20 ± 4.8	5	1.6		<0.04	5	<0.60	5	-
Yesso scallop	20 May 2025	4.6 ± 0.95	5	135 ± 43	5	3.4		<0.04	5	<0.60	5	-
Yesso scallop	17 June 2025	2.5 ± 0.35	5	104 ± 21	5	2.4		<0.04	5	<0.60	5	-
Yesso scallop	21 October 2025	1.4 ± 0.34	5	8.2 ± 0.94	5	17		0.075 ± 0.035	5	1.4 ± 0.50	4	5.4
Akazara scallop	28 August 2025	<0.06	1	23	1	-		0.045	1	10	1	0.5
Akazara scallop	27 January 2026	<0.06	5	5.0 ± 2.4	5	-		0.16 ± 0.10	5	3.3 ± 2.1	5	4.8

The LOD values of TTX in hemolymph-S and digestive gland were 0.04 nmol/mL and 0.60 nmol/g, respectively. The LOD value of total PSTs was 0.06 nmol/mL.

## Data Availability

Data are available only in this article.

## Supplementary Materials

The following supporting information can be downloaded at: https://www.mdpi.com/article/10.3390/md24060200/s1, Figure S1: Representative LC-FLD chromatograms of standards and samples, C1/C2, GTX1-5, and STXs; Figure S2: Representative LC-FLD chromatograms of TTX; Figure S3: Representative LCMS (MRM) mass chromatograms of TTX; Figure S4: LCMSMS spectra of TTX in the hemolymph-S in Yesso scallop (21 October 2025), TTX in the hemolymph-S in Akazara scallop (28 August 2025), and TTX standard; Figure S5: Comparison of PSTs composition between intact and acidified-heated hemolymph-S; Table S1: PSTs concentrations in digestive gland and hemolymph-S in Yesso scallops collected at four times; Table S2. Size and weight of individual scallop samples. References [49,50,51,52,53] are cited in the Supplementary Materials.

## Abbreviations

The following abbreviations are used in this manuscript:

PSTs	Paralytic shellfish toxins
TTX	Tetrodotoxin
STX	Saxitoxin
dcSTX	Decarbamoyl saxitoxin
GTX	Gonyautoxin
Hemolymph-S	Supernatant of hemolymph
LC-FLD	Liquid chromatography-fluorescence detection
HILIC	Hydrophilic interaction liquid chromatography
LCMS	Liquid chromatography mass spectrometry
HR	High resolution
LOD	Limit of detection
LOQ	Limit of quantification
AcOH	Acetic acid
EtOH	Ethanol
MeCN	Acetonitrile
MRM	Multiple reaction monitoring
EIC	Extracted ion chromatogram
