# Supplementary material for: Hemolymph-Mediated Dynamics of Paralytic Shellfish Toxins and Tetrodotoxin in Scallops
Source: Mar Drugs. 2026 Jun 5;24(6):200. doi: 10.3390/md24060200 (PMC13301539; doi:10.3390/md24060200)
Supplement: Supplementary file 1 [file marinedrugs-24-00200-s001.zip › marinedrugs-4327975-supplementary.pdf]

Table S1. Quantitative data of PSTs in digestive gland and hemolymph-S in Yesso scallops collected at four times

	digestive gland		nmol/g			hemolymph-S	nmol/mL	
		n	mean	SD		n	mean	SD
22 April 2025	STX	5	0.82	0.30		5	<0.011	
	neoSTX	5	0.04	0.06		5	<0.005	
	dcSTX	5	1.13	0.36		5	<0.010	
	GTX1	5	0.21	0.15		5	0.043	0.058
	GTX2	5	0.09	0.07		5	0.010	0.012
	GTX3	5	0.06	0.05		5	0.014	0.018
	GTX4	5	0.40	0.41		5	0.093	0.127
	GTX5	5	0.07	0.07		5	<0.016	
	C1	5	1.47	0.68		5	0.029	0.016
	C2	5	0.97	0.45		5	0.154	0.082
	digestive gland		nmol/g			hemolymph-S	nmol/mL	
20 May 2025		n	mean	SD		n	mean	SD
	STX	5	1.47	1.2		5	<0.011	
	neoSTX	5	5.99	5.7		5	0.15	0.10
	dcSTX	5	0.50	0.5		5	<0.010	0.00
	GTX1	5	41.09	43.4		5	0.63	0.27
	GTX2	5	16.94	12.9		5	0.19	0.09
	GTX3	5	10.69	7.6		5	0.30	0.16
	GTX4	5	16.16	21.0		5	2.17	0.90
	GTX5	5	<0.016			5	<0.016	
	C1	5	9.80	7.2		5	0.18	0.08
	C2	5	6.34	4.5		5	0.94	0.44
	digestive gland		nmol/g			hemolymph-S	nmol/mL	
17 June 2025		n	mean	SD		n	mean	SD
	STX	5	1.90	2.95		5	<0.011	
	neoSTX	5	4.71	11.34		5	<0.005	
	dcSTX	5	0.44	0.49		5	<0.010	
	GTX1	5	36.96	11.37		5	0.71	0.09
	GTX2	5	23.19	7.41		5	0.71	0.33
	GTX3	5	8.69	3.79		5	0.26	0.13
	GTX4	5	13.75	8.05		5	0.34	0.11
	GTX5	5	1.85	0.14		5	<0.016	
	C1	5	3.43	2.22		5	0.22	0.15
	C2	5	0.87	0.37		5	0.19	0.10
	digestive gland		nmol/g			hemolymph-S	nmol/mL	
21 October 2025		n	mean	SD		n	mean	SD
	STX	5	0.33	0.09		5	<0.011	
	neoSTX	5	0.83	0.28		5	<0.005	
	dcSTX	5	0.00	0.00		5	<0.010	
	GTX1	5	0.82	0.46		5	0.32	0.18
	GTX2	5	2.73	0.47		5	0.68	0.27
	GTX3	5	1.94	0.43		5	0.24	0.10
	GTX4	5	0.86	0.37		5	0.15	0.06
	GTX5	5	0.59	0.17		5	<0.016	
	C1	5	0.05	0.02		5	0.01	0.003
	C2	5	0.02	0.01		5	0.001	0.0004

Table S2. Size and weight of scallop samples

Yesso Scallop — 22 April 2025

	length (cm)	wide (cm)	edible tissue (g)	digestive gland (g)
no.1	10.5	13	82	7.4
no.2	12	12.5	75	7.7
no.3	11.5	12.5	72	7.4
no.4	12	12.6	106	7.9
no.5	11.8	12.7	91	9.2
minimum–maximum	10.5-12.0	12.5-13.0	72-106	7.4-9.2

Yesso Scallop — 20 May 2025

	length (cm)	wide (cm)	edible tissue (g)	digestive gland (g)
no.1	13.3	13.6	107	11
no.2	12.3	12.7	110	12
no.3	12.5	12.7	87	11
no.4	11.8	12.9	131	12
no.5	13.1	13.7	131	13
minimum–maximum	11.8-13.3	12.7-13.7	87-131	11から13

Yesso Scallop — 17 June 2025

	length (cm)	wide (cm)	edible tissue (g)	digestive gland (g)
no.1	11.7	12.6	93	7.9
no.2	12.3	12.7	82	7.3
no.3	12	12.6	84	8.6
no.4	13.1	13.3	104	10.6
no.5	11.9	13.1	109	9.2
minimum–maximum	11.7-13.1	12.6-13.3	82-109	7.3-10.6

Yesso Scallop — 21 October 2025

	length (cm)	wide (cm)	edible tissue (g)	digestive gland (g)
no.1	13.2	14.3	97	
no.2	13.5	14.6	97	
no.3	13.7	14.3	80	not determined
no.4	14.1	14.7	130	
no.5	14	15.5	122	
minimum–maximum	13.2-14.1	14.3-15.5	80-130	

Akazara scallop — 28 August 2025

	length (cm)	wide (cm)	edible tissue (g)	digestive gland (g)
no.1	9	8.6	32	1.5
no.2	7.6	7.5	26	0.72

Akazara scallop — 27 January 2026

	length (cm)	wide (cm)	edible tissue (g)	digestive gland (g)
no.1	8.3	7.8	30	2.3
no.2	8.5	8	34	2.6
no.3	7.5	7.2	31	2.5
no.4	8.6	8.3	40	2.8
no.5	9.3	8.9	49	3.4
minimum–maximum	7.5-9.3	7.2-8.9	30-49	2.3-3.4

Figure S1
Representative LC-FLD chromatograms o
standards and samples
C1/C2, GTX1-5, and STXs
Sample: Yesso scallop
22 April 2025 no.1

Condition:
Oshima, Y., Postcolumn Derivatization Liquid Chromatographic Method for Paralytic Shellfish Toxins. *J. AOAC Int.* **1995,** 78, (2), 528–532.

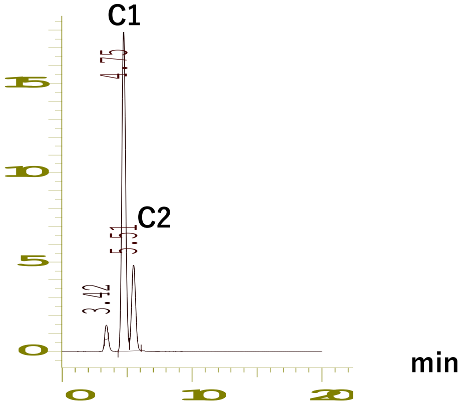

Fig. S1-1 C1/2 standard (TUKC9112 (1/50))

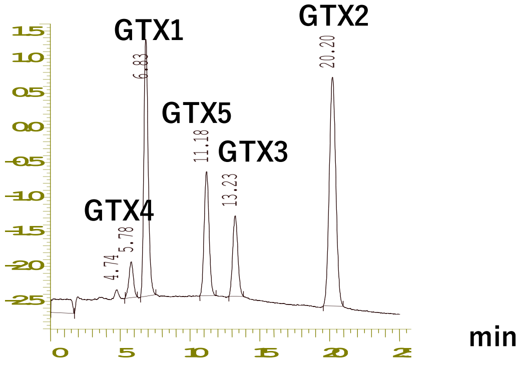

Fig. S1-4 GTX1-5 standard (TUKC9112 (1/50))

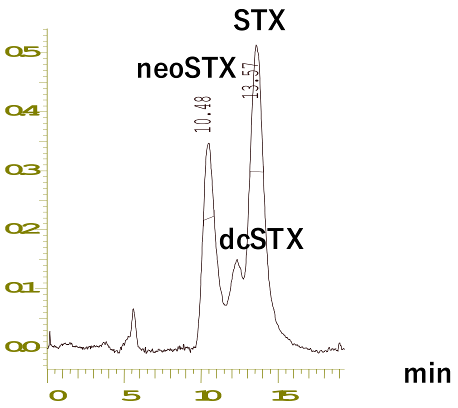

Fig. S1-7 neoSTX, dcSTX, STX standard
(TUMS913 (1/5) #10)

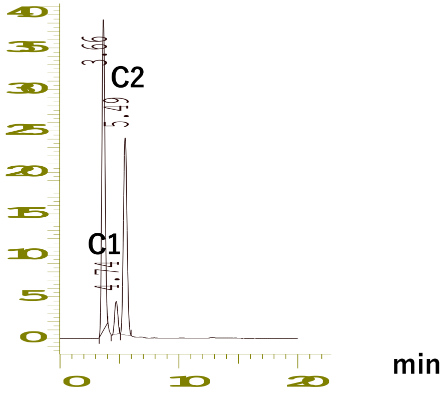

Fig. S1-2 *M. yessoensis* no.1
Hemolymph-S C1/C2

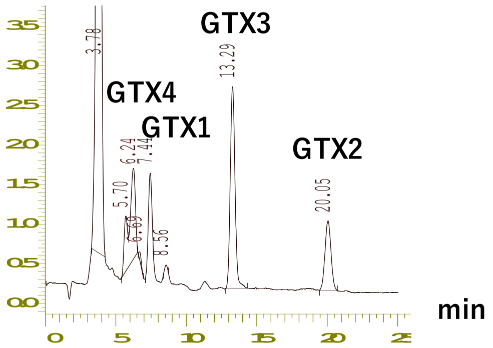

Fig. S1-5 *M. yessoensis* no.1
Hemolymph-S G-toxins

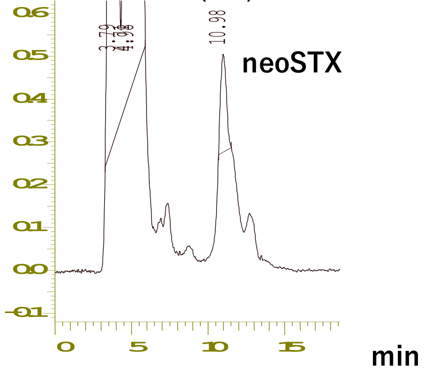

Fig. S1-8 *M. yessoensis* no.1
Hemolymph-S S-toxins

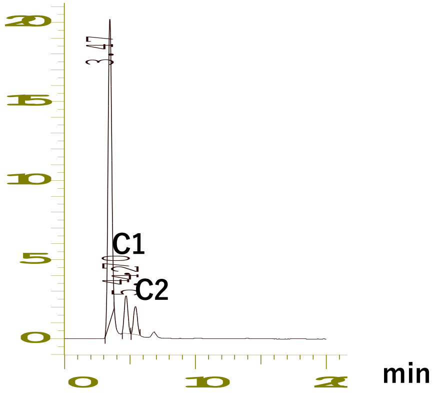

Fig. S1-3 *M. yessoensis* no.1
Digestive gland C1/C2

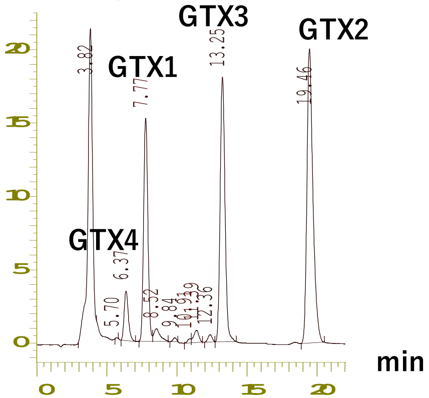

Fig. S1-6 *M. yessoensis* no.1
Digestive gland G-toxins

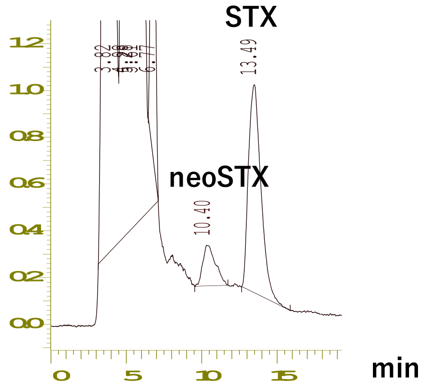

Fig. S1-9 *M. yessoensis* no.1
Digestive gland S-toxins

Figure S2. Representative LC-FLD chromatograms of TTX

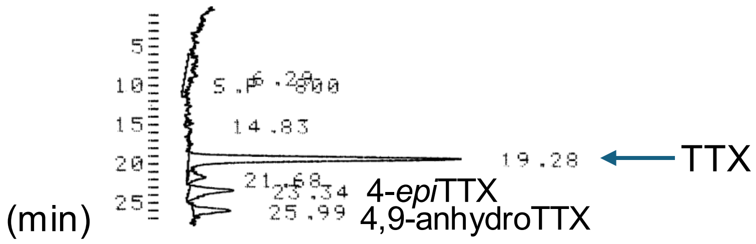

Standard TTX 5ng

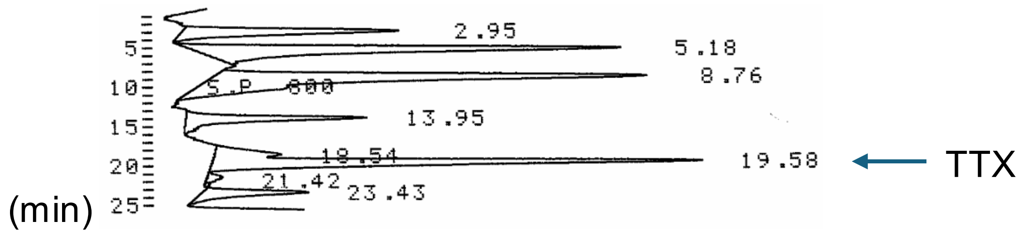

Digestive gland of *C. farreri akazara*
27 January 2026 no.5

Column: Develosil C30 UG-5 (5 µm, i.d. 0.46 x 15 cm), LC solvent: An aqueous solution containing 1 vol% acetonitrile, 20 mM ammonium heptafluorobutyrate, and 10 mM ammonium formate buffer (pH 5.0) at a flow rate of 0.4 ml/min using a pump Hitachi L-6000. The eluted compounds were heated with 4 N NaOH (flow rate 0.7 ml/min, pump Hitachi L7100) at 105° C in a stainless tube (i.d. 0.46 mm x 5 m). The reaction products were detected by a Jasco FP2025 fluorescence detector with excitation 365 nm and emission 510 nm. A Hitachi D-2500 Chromato-Integrator was utilized for data acquisition and plotting. See ref. 52,53 in text.

Figure S3. Representative LCMS (MRM) mass chromatograms of TTX

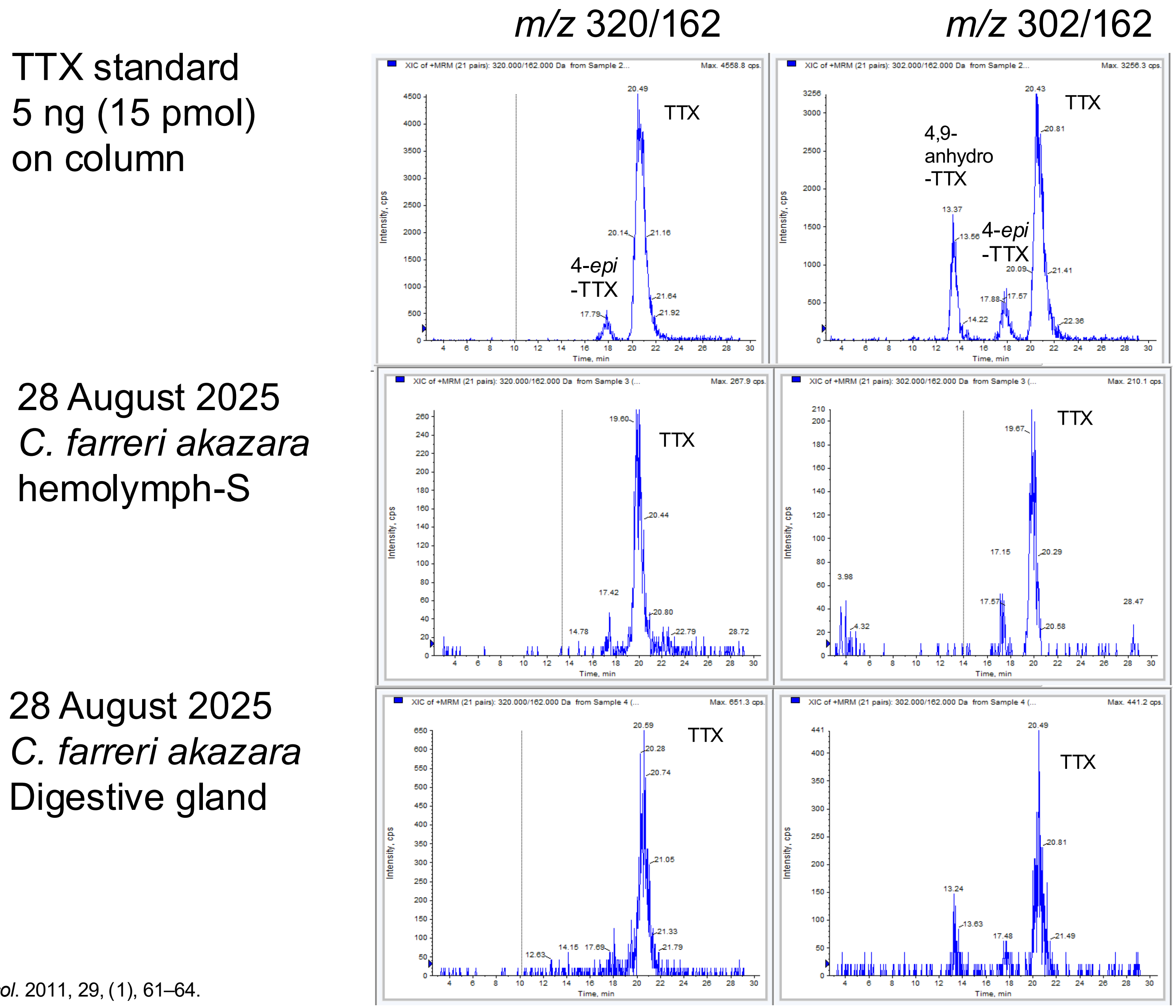

Condition: Yotsu-Yamashita, M. et al., *Forensic Toxicol.* 2011, 29, (1), 61–64.

Figure S4. LCMSMS spectra of TTX in the hemolymph-S in Yesso scallop (21 October 2025), the hemolymph-S in Akazara scallop (28 August 2025), and standard TTX (1 ng on column).

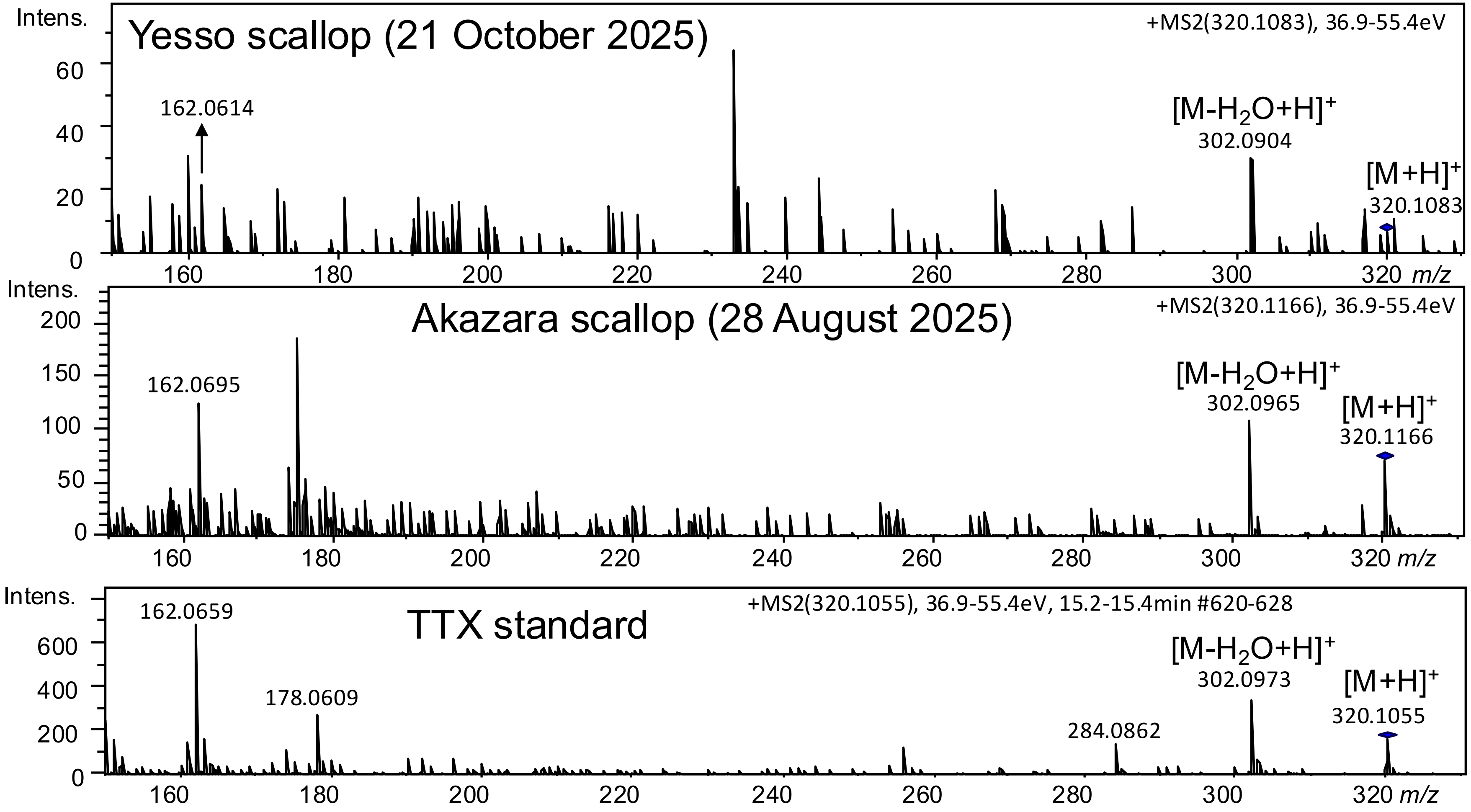

TTX in Yesso scallop (21 October 2025) : Precursor ion : *m/z* 320.1083
The product ion at *m/z* 302.0904 is the dehydrated ion from TTX, and the ion at *m/z* 162.0614 is the characteristic product ion of TTX as we reported previously (Yotsu-Yamashita, M. et al., *Mar. Drugs*, 2013, 11, 2799-2813).

Figure S5. Comparison of PSTs composition between intact hemolymph-S and acidified and heated hemolymph-S

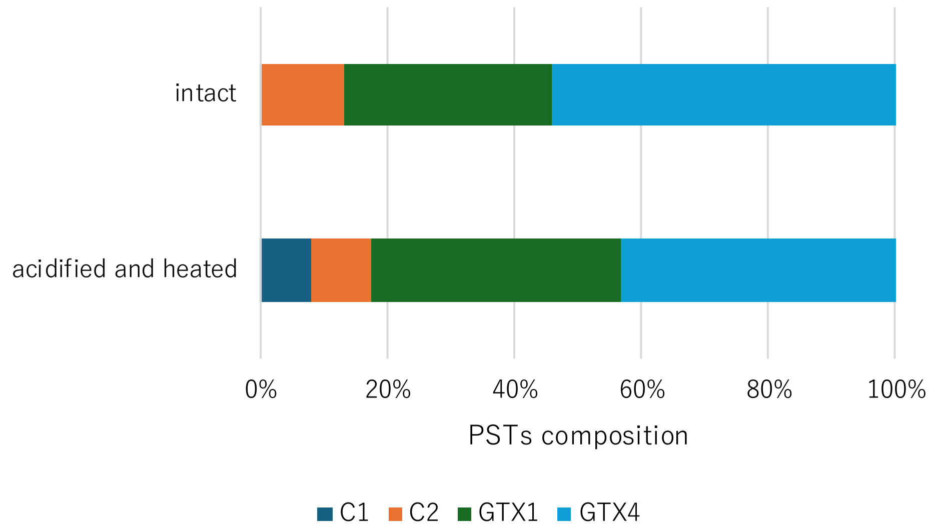

20 May 2025 hemolymph-S sample (1 mL) was acidified with AcOH (1% AcOH, v/v) and heated in the boiling water for 5 min. After cooling and neutralization of the sample solution with 2.5% NH_4OH, PSTs were purified with activated charcoal (1.5 mL) as described in the text. The same hemolymph-S sample (intact) was directly treated with activated charcoal without acidifying and heating. In both samples, PSTs were quantified using HR-HILIC-LCMS (n=1). Partial transformation from C2 to C1 and GTX4 to GTX1 were indicated, while transformation from C1/C2 to GTX 2/3 (hydroxylation of carbamoyl N-SO_3) was not suggested. GTX 2/3, STX, neoSTX, and dcSTX were not detected in both. The data of hemolymph-S shown in the text was all obtained without acidifying and heating.
